# CE Neuropsychological and neurobehavioral outcome following childhood arterial ischemic stroke: Attention deficits, emotional dysregulation, and executive dysfunction

**DOI:** 10.1080/09297049.2013.832740

**Published:** 2013-09-12

**Authors:** Fiadhnait O'Keeffe, Frédérique Liégeois, Megan Eve, Vijeya Ganesan, John King, Tara Murphy

**Affiliations:** 1 Research Department of Clinical, Educational and Health Psychology, University College, London, London, UK; 2 Institute of Child Health, University College London, London, UK; 3 Department of Clinical Neuropsychology, Great Ormond Street Hospital for Children NHS Foundation Trust, London, UK

**Keywords:** Childhood arterial ischemic stroke, Neuropsychology, Neurobehavior, Attention, Executive function, Emotional regulation

## Abstract

**Objectives:**

To investigate neuropsychological and neurobehavioral outcome in children with arterial ischemic stroke (AIS).

**Background:**

Childhood stroke can have consequences on motor, cognitive, and behavioral development. We present a cross-sectional study of neuropsychological and neurobehavioral outcome at least one year poststroke in a uniquely homogeneous sample of children who had experienced AIS.

**Method:**

Forty-nine children with AIS aged 6 to 18 years were recruited from a specialist clinic. Neuropsychological measures of intelligence, reading comprehension, attention, and executive function were administered. A triangulation of data collection included questionnaires completed by the children, their parents, and teachers, rating behavior, executive functions, and emotions.

**Key Findings:**

Focal neuropsychological vulnerabilities in attention (response inhibition and dual attention) and executive function were found, beyond general intellectual functioning, irrespective of hemispheric side of stroke. Difficulties with emotional and behavioral regulation were also found. Consistent with an “early plasticity” hypothesis, earlier age of stroke was associated with better performance on measures of executive function.

**Conclusions:**

A significant proportion of children poststroke are at long-term risk of difficulties with emotional regulation, executive function, and attention. Data also suggest that executive functions are represented in widespread networks in the developing brain and are vulnerable to unilateral injury.

Special appreciation to all the children, parents, and their teachers without whose generous participation this research would not have been possible. This research was partially funded by The University of London Central Research Fund, the Graduate School University College London, and The Research Department of Clinical, Educational and Health Psychology University College London to the first author (F. O'Keeffe). No conflict of interest exists.

Stroke in childhood can result in significant residual physical and cognitive impairments in two thirds of survivors (Cnossen et al., 2010; Steinlin, Roelin, & Schroth, 2004). The vast majority of studies of childhood stroke are heterogeneous in terms of etiologies included and thus there is limited potential to explore the correlates of subgroups with similar lesion patterns. The current study focuses specifically on childhood arterial ischemic stroke (AIS). AIS is an acute focal neurological deficit attributable to cerebral infarction in an arterial distribution and affects approximately 3 per 100,000 children a year—an incidence rate as frequent as children with brain tumors (Jordon, 2006). Experiencing an ischemic stroke during childhood has been shown to significantly lower a young person's quality of life across physical, emotional, school, social, and cognitive areas (O'Keeffe, Ganesan, King, & Murphy, 2012). There is a dearth of research investigating the longer term neuropsychological and neurobehavioral sequelae following childhood AIS. This research is fundamental to the development of appropriate interventions and programs for children who have experienced childhood stroke and their families.

Recent neuropsychological studies have indicated that few domains of cognitive functioning are unaffected following childhood stroke. Studies that have focused specifically on the impact of stroke on a child's general intellect have shown that group mean intelligence quotient scores tend to fall in the lower end of the average range (i.e., Full Scale IQ [FSIQ] between 90 and 95) but significantly lower than control groups or standardized population norms (Everts et al., 2008; Max et al., 2002; Pavlovic et al., 2006; Westmacott et al., 2009). Few studies have specifically investigated attention and executive function abilities following childhood stroke (Long, Anderson, et al., 2011). Furthermore, of the small number of studies that do exist, ischemic, hemorrhagic, and venous strokes are commonly included together, limiting the conclusions that can be drawn (e.g., Long, Spencer-Smith, et al., 2011; and Long, Anderson, et al., 2011, ischemic and hemorrhagic; Pavlovic et al., 2006, ischemic and venous; Max et al., 2002, ischemic and hemorrhagic).

Difficulties with sustained and divided attention, visual search, decreased accuracy, and increased variability in reaction times are reported (Everts et al., 2008; Long, Anderson, et al., 2011; Max et al., 2003, 2004; Schatz et al., 1999). Speed of information processing also appears consistently reduced and increased cognitive effort is required, particularly where accurate performance is maintained (Block, Nanson, & Lowry, 1999). Previous research examining attention has used experimental paradigms adopting reaction time and variability performance measures for alertness, divided attention, and visual search/orientation (Max et al., 2004). Few studies reporting attention difficulties in children with stroke have used measures that are easily transferable to clinical practice. Working memory appears vulnerable, as demonstrated by specific tasks, such as the Digit Span subtest from the Wechsler Intelligence Scales (Everts et al., 2008; Wechsler, 1991, 2004; Westmacott et al., 2009; White, Salorio, Schatz, & DeBaun, 2000). A recent study showed that irrespective of lesion location (frontal, extra-frontal, cortical, or subcortical), children with stroke demonstrated significant impairment on clinical measures of executive function, including attentional control, cognitive flexibility, goal setting, and information processing (Long, Spencer-Smith, et al., 2011). Larger lesions were associated with greater executive dysfunction (Long, Anderson, et al., 2011). These studies supported the view of widespread, diffuse but integrated functional representation of executive functions in the developing brain. They also demonstrated that integrity of the entire brain and vasculature is essential for the normal development of executive function skills. However, heterogeneity of type of stroke (ischemic, hemorrhagic, venous) existed in several of these studies (Long, Anderson, et al., 2011; Long, Spencer-Smith et al., 2011; Max et al., 2002, 2003, 2004; Pavlovic et al., 2006).

Behavior and emotional regulation is an area of difficulty commonly reported following childhood stroke, with 33% to 59% of parents reporting concerns (Ganesan et al., 2000; Pavlovic et al., 2006; Steinlin et al., 2004). Max et al. (2002) found that 59% of the children in their study fulfilled diagnostic criteria for a psychiatric disorder (attention deficit/hyperactivity disorder [46%]; anxiety disorders [31%]; mood disorders [21%]) with frequent comorbidity. Children with ischemic and hemorrhagic strokes have also been shown to have more difficulties in everyday executive function behaviors, including behavioral regulation and metacognitive abilities, as rated by their parents and teachers on the Behavioral Rating Inventory of Executive Function (BRIEF; Gioia, Isquith, Guy, & Kenworthy, 2000; Long, Anderson, et al., 2011). Williams et al. (2012) found that children with moyamoya vasculopathy were at risk for both intellectual and executive function difficulties, as measured by parent- and teacher-rated BRIEFs. However, children's own ratings of their awareness of behavioral and emotional difficulties have not been reported. The current study is the first to address this triangulation of data from children themselves, their parents, and their teachers.

There is ongoing debate and controversy around two competing views in the literature: that of early brain plasticity (Ballantyne, Spilkin, Hesselink, & Trauner, 2008) versus the early vulnerability hypothesis (Anderson et al., 2009, 2010). The debate has found mixed and inconsistent support from research in neonatal and childhood stroke. Studies have reported that younger age of stroke onset was associated with poorer functional outcome (Ganesan et al., 2000) and with more severe neurological outcome disability ratings at follow-up (Cnossen et al., 2010). There is evidence that the effect of age of stroke onset on outcome may be task dependent. Long, Anderson, et al. (2011) reported that early onset ischemic and hemorrhagic stroke survivors (stroke < 5 years) performed more poorly on some aspects of executive function, including attentional control, but better on others, such as goal setting. Max, Bruce, Keatley, and Delis (2010) found that the differences between an early onset ischemic and hemorrhagic group (stroke < 12 months) and matched controls were larger than the differences between their late-onset group (stroke > 12 months) and matched controls on many cognitive tasks. However, there were larger differences between late onset and controls on two executive function tasks, suggesting that for some cognitive tasks, such as executive functions, later age of stroke may be a disadvantage. Allman and Scott (2013) found that children who experienced ischemic stroke under the age of 1 *and* after the age of 6 performed more poorly on neuropsychological assessment, suggesting a nonlinear effect of age at stroke. Methodological limitations complicate these findings, such as differing classification of “early” versus “late” onset stroke and inclusion of mixed stroke type in several studies. Several previous studies also include neonatal, perinatal, and childhood stroke (e.g., Max et al., 2004; Pavlovic et al., 2006; Westmacott et al., 2009). Studies with small sample sizes reduce power further by dividing into subcategories of age of stroke onset. Unlike previous studies, and in recognition that it may be preferable to analyze age as a continuous variable (Taylor & Alden, 1997), we chose to explore the effect of age at stroke using correlational analysis, rather than by subdividing our sample into several groups.

This study describes the largest homogeneous cohort of childhood AIS, excluding neonatal or perinatal stroke. This is a homogeneous cohort of AIS, as the majority of children had basal ganglia and/or middle cerebral artery (MCA) territory AIS. We also uniquely adopted a triangulated approach of self, parent, and teacher ratings of everyday executive function and behavioral and emotional functioning. Due to the large sample size, the results enable us to explore the effects of age at stroke on attention, executive function, and neurobehavioral outcome. There has been a call for the inclusion of measures of neurobehavior, academic attainment, and executive function in assessments of outcome (Long, Anderson, et al., 2011; Taylor & Alden, 1997). This study therefore aims to investigate key areas of likely cognitive, behavioral, and emotional difficulty, including general intelligence, academic attainment, attention, executive function, and behavior. In this study, a subset of 9 participants were followed up at 19–31 months in order to investigate the longitudinal impact of childhood AIS.

We hypothesize that (a) following AIS, children will have particular difficulties with attention and executive function skills on performance-based assessment and behavioral ratings, while performance on general intellectual functioning and academic attainment will be maintained within the average range; (b) following AIS, there will be no lateralization effects noted on assessments; (c) younger age of AIS will be associated with poorer outcome on assessments of general intellectual functioning, academic attainment, and executive functions.

## METHOD

### Participants

Full ethical approval was received from the National Health Service Research Ethics Committee. Children were recruited from a specialist pediatric neurovascular clinic in London, United Kingdom. This clinic serves to follow up all children with cerebrovascular disorders presenting from the referral population of the hospital (North London). Inclusion criteria were (a) aged between 6–18 at assessment, (b) experienced an Arterial Ischemic Stroke (AIS) beyond the neonatal/perinatal period, that is, beyond 28 days of life, (c) MCA territory AIS, and (d) English speakers.

Sixty-four children met the inclusion criteria. Forty-nine children agreed to participate and were assessed either in an outpatient clinic or in their homes between August 2009 and February 2010. The group comprised of 30 boys (61.2%) and 19 girls (38.8%), ranging between 6 and 18 years at assessment (*M* = 11.08, *SD* = 3.65). Mean time since stroke onset was 6 years (*SD* = 3.41). Demographic and clinical characteristics of the participants are presented in Tables 1 and 2. As can be seen in Table 2, the majority of ischemic strokes included basal ganglia and/or MCA infarcts, with the frontal cortical areas predominantly spared.

**Table 1.** Demographics and Clinical Characteristics of the Sample.

Sample characteristics	*n* (%)/ Mean (*SD*)
*N*	49
Sex, *n* (%) males	30 (61.2%)
SES (NS-SEC) Mean (*SD*)	2.65 (1.81)
SES 1: Management/Professional	23 (50%)
SES 2: Intermediate	2 (4.3%)
SES 3: Small employers	3 (6.5%)
SES 4: Lower supervisory/Technical	4 (8.7%)
SES 5: Routine/Unemployed	14 (30.4%)
Ethnicity, *n* (%)	
White British	33 (67.3%)
Black African	4 (8.2%)
Black Caribbean	4 (8.2%)
Asian	5 (10.2%)
White European	3 (6.1%)
Age at stroke onset Mean (*SD*; Range)	5.08 (SD 3.67) (Range 4 mths–15.66 yrs)
Age at assessment Mean (*SD*; Range)	11.08 (SD 3.65) (Range 6.0 yrs–18.4 yrs)
Time since stroke onset Mean (*SD*; Range)	6.0 (SD 3.41) (Range 7 mths–15.26 yrs)
Etiology/ Identified risk factors: *n* (%)	
Sickle Cell Disease	7 (14.3%)
Moyamoya	10 (20.4%)
Chicken pox/Other infection (e.g. shingles)	11 (22.4%)
Cerebrovascular abnormality identified	9 (18.4%)
Cardiac abnormality identified	3 (6.1%)
Other (e.g., Dissection)	5 (10.2%)
Unknown/None identified	4 (8.2%)
Neurological Severity Motor Score Mean (*SD*)	1.73 (.81)
1: Normal or only reflex asymmetry, *n* (%)	24 (49%)
2: Mild hemiparesis, can do isolated finger movements, *n* (%)	14 (28.6%)
3: Severe hemiparesis, cannot do isolated finger movements, *n* (%)	11 (22.4%)
Lateralization of stroke, *n* (%):	
Left	23 (46.9%)
Right	21 (42.9%)
Bilateral	5 (10.2%)
Handedness (Right), *n* (%)	29 (61.7%)
Changed handedness since stroke, *n* (%)	18 (40.9%)
Recurrent stroke or TIAs, *n* (%)	27 (55.1%)
History of seizures, *n* (%)	13 (26.5%)
Currently taking Antiepileptic Drugs, *n* (%)	7 (14.3%)
Education:	
Statement of Special Education Needs, *n* (%)	10 (20.8%)
Special Education Register, *n* (%)	28 (58.3%)
Extra help in school, hours, Mean (*SD*)	4.05 (6.69)

*Notes. SD* = Standard Deviation; TIA = Transient Ischemic Attack; SES = Socioeconomic Status; NS-SEC: National Statistical Socio-Economic Classification Self-Coding Method (Office for National Statistics, 2005).

**Table 2:** Clinical Characteristics of Participants.

Participant	Sex	Age (in years) at assessment	Age (in years) at stroke onset	Clinical Course/ Presentation	Vascular territory of initial stroke	Any recurrent TIAs or Stroke	Diagnoses/ Risk factors	Seizures
1	F	6.75	3.08	R hemiparesis	L MCA	N	None identified	N
2	M	6.33	1.42	L hemiparesis	R MCA	N	Chicken pox	N
3	F	11.60	6.33	L hemiplegia	R MCA	N	Cerebrovascular abnormality	N
4	M	8.00	1.33	R hemiparesis, aphasia	L MCA BG	N	Chicken-pox	N
5	F	12.00	10.66	L hemiplegia Headache	R ICA and MCA	N	Dissection of the right internal carotid artery	N
6	M	10.50	2.25	L face weakness	R MCA BG	Y	Chicken Pox	N
7	F	8.00	4.50	R hemiplegia Pain R facial palsy	L BG MCA	N	None identified	N
8	M	16.83	6.25	Headache L hand weakness	R fronto/temporal	Y	Cardiac-related	N
9	F	9.08	2.50	L sided weakness	R BG/MCA	Y	Moyamoya	Y
10	M	9.25	4.25	R hemiparesis, facial involvement, aphasia	L MCA	N	Chickenpox	N
11	F	8.25	5.25	R hemiparesis, dizziness, dysarthria, disturbed speech	L MCA	?	Chickenpox	N
12	M	8.08	5.42	L sided weakness, Headache Pain Paraesthesiae Dysarthria Ataxia	R parietal, R MCA, and Multiple posterior circulation territory infarcts	Y	Cerebrovascular abnormality	N
13	F	17.50	12.42	R hemiplegia and pseudobulbar palsy R homonymous hemianopia	L MCA	Y	Other Pre-existing mild-moderate Learning Disability	Y
14	M	12.16	6.92	L sided weakness Drooling Facial palsy	R BG MCA infarct	Y	Chicken pox	N
15	M	16.42	1.16	L hemiparesis	R BG MCA infarct	N	Moyamoya	N
16	M	17.50	13.66	L sided weakness and facial droop	R MCA	Y	Sickle cell disease	Y
17	F	8.25	3.42	L hemiparesis	Bilateral/Diffuse	Y	Moyamoya	N
18	M	11.66	1.08		R MCA R temporal frontal infarct		Cardiac-related	
19	M	6.08	1.00	L sided stiffening	R BG MCA	N	Cerebrovascular abnormality	Y
20	M	12.83	10.08	R sided weakness and slurred speech R facial weakness	L MCA territory stroke	N	Cerebrovascular abnormality	N
21	M	13.58	1.08	R Hemiparesis	L BG MCA infarct	N	Herpes Zoster vasculopathy Cerebrovascular disease	N
22	M	14.00	12.92	R arm stiffening	L frontal lobe and MCA	Y	None identified	N
23	M	15.25	8.00	Headache R weakness Dysarthria R facial palsy	L BG MCA	N	Cerebrovascular abnormality	N
24	F	6.00	5.42	Headache, R sided weakness, drooling, slurred speech Vomiting	L MCA	N	Other- Trauma 4 days prior	N
25	M	14.00	6.08	R sided facial weakness R upper and lower limb weakness Slurred speech	L BG MCA and cortical	N	Chicken Pox	N
26	F	6.83	3.25	Acute R hemiparesis in upper and lower limb	L fronto-parietal cortex, bilateral MCA	? TIAs	Moyamoya	
27	M	6.25	4.50	Acute R sided weakness Dysarthria	L MCA infarct	Y	Sickle cell disease	
28	F	8.50	1.66	R hemiparesis	L frontal	Y	Moyamoya	Y
29	F	11.00	9.50	R hemiplegia, facial nerve palsy Headache L ptosis	L MCA	Y	Other- Intracranial arterial dissection	N
30	M	7.75	0.83	Dystonic L hemiparesis	R BG MCA		Chicken Pox	
31	M	12.66	1.25	L hemiparesis	R BG MCA		Chicken pox	
32	F	9.42	3.00	L hemiplegia	R frontotemporal region	Y	Sickle cell disease	N
33	F	6.25	2.58	L sided weakness Vomiting Fever	R MCA distribution	N	Cardiac-related	N
34	M	16.5	4.58	Seizures	L fronto-parietal region	Y	Moyamoya	Y
35	M	10.92	2.50	Fatigue, L sided facial and upper limb weakness	R BG MCA	N	Cerebrovascular abnormality	
36	M	11.16	2.92	R hemiparesis and aphasia	L BG MCA	Y	Cerebrovascular abnormality	Y
37	F	12.66	7.66	Seizures	L MCA	N	Sickle cell disease	Y
38	M	11.50	6.00	R hemiparesis arm and leg	L frontal	Y	Sickle cell disease	
39	F	13.92	7.00	Sensory symptoms L sided hemiparesis	Bilateral/ Diffuse	Y	Moyamoya	
40	M	12.92	6.75	L sided weakness hemiparesis Behavior change	R ACA and MCA	Y	Sickle Cell	Y
41	F	10.92	5.00	Headaches	Bilateral/Diffuse	−	Moyamoya	−
42	M	17.00	3.00	Dystonic L hemiparesis	R BG infarct MCA	Y	Cerebrovascular abnormality	−
43	M	18.42	15.66	L hemiplegia Slurred speech Parasthesia of L leg	R MCA	N	Other- Stage 4 Neuroblastoma-	N
44	M	10.17	4.83	Expressive dysphasia, Weakness on L	Bilateral/ Diffuse	Y	Moyamoya	Y
45	M	17.25	8.33		Bilateral/Diffuse	Y	Sickle Cell Disease	N
46	M	6.00	3.25	L hemiplegia arm Slurred speech	R MCA	N	Chicken pox	N
47	F	8.17	6.58		L	−	None identified	−
48	M	8.75	0.33	R sided hemiplegia R sided facial weakness and drooling.	L MCA	N	Cerebrovascular abnormality	Y
49	F	8.25	1.50	R sided partial seizures.	L	Y	Moyamoya	Y

*Notes.* L = Left; R = Right; MCA = Middle Cerebral Artery; BG = Basal Ganglia; Y = Yes; N = No.

Motor impairment was rated as (a) absent, (b) mild (hemiparesis, able to isolate individual finger movements), or (c) severe (hemiparesis, unable to isolate finger movements). The neurological severity ratings indicated that half the sample (49%) showed normal or reflex asymmetry only, and 51% showed hemiparesis (weakness on one side of the body), either mild (able to do isolated finger movements) or severe (unable to do isolated finger movements). The most common risk factors for AIS identified were chicken pox/other infections, moyamoya, and other cerebrovascular abnormalities. Over half the participants had recurrent transient ischemic attacks or recurrent stroke, and a quarter had a history of seizures.

A subgroup (*n* = 9) was followed up at 19–31 months since initial assessment. Follow-up assessments were conducted in participant homes between November 2011 and March 2012. The follow-up group comprised of 4 boys (44%) and 5 girls (56%), aged between 10 and 19 years (*M* = 14.43, *SD* = 2.90).

561

### Measures

**General Intellectual Ability.** The Wechsler Abbreviated Scale of Intelligence (WASI; Wechsler, 1999) was administered to all children. Two subtests from the Wechsler Intelligence Test for Children, 4th edition (WISC-IV; Wechsler, 2004), Digit-Span (to measure auditory working memory/ attention) and Coding (to measure processing speed), were also administered, given previous findings of vulnerabilities in these areas. For teenagers aged 17 and 18, the equivalent subtests (Digit Span and Digit-Symbol Coding) of the Wechsler Adult Intelligence Test, 3rd edition (WAIS-III; Wechsler, 1997) were administered.

**Academic Attainment.** The Reading Comprehension subtest of the Wechsler Individual Achievement Test, 2nd Edition (WIAT-II UK; Wechsler, 2005) was administered. Reading Comprehension is seen as an important area of academic attainment that is a more complex and higher order academic skill than single-word reading. Reading Speed, as calculated by time taken in seconds to read each section of the reading comprehension subtests, was also compared to standardized norms.

**Attention.** The Test of Everyday Attention for Children (TEA-Ch; Manly, Robertson, Anderson, & Nimmo-Smith, 1998) is a standardized test of attention, suitable for children and young persons between the ages of 6–16 years. Five subtests of the TEA-Ch were administered: Sky Search, Score, Sky Search Dual Task, Score Dual Task, and Walk/Don't Walk.

**Executive Function.** The Trail-Making Test (TMT) of the Delis-Kaplan Executive Function System (D-KEFS; Delis, Kaplan, & Kramer, 2001) was administered. It is suitable for children and adults between the ages of 8 and 89.

The Behavior Rating Inventory of Executive Function (BRIEF; Gioia et al., 2000) was administered to children, parents, and teachers. Norms are available for ages 5–18 years for the parent and teacher versions. The child self-report version is available for children and young persons aged 11–18 years. The BRIEF yields an index of Behavioral Regulation and of Metacognition and an overall Global Executive Composite Score (Mean *T* = 50, *SD* = 10). Higher *T*-scores are indicative of greater difficulties.

**Behavior.** The Strengths and Difficulties Questionnaire (SDQ; Goodman, 1997) is a 25-item brief behavioral screening questionnaire yielding five scales: Emotional Symptoms, Behavior Problems, Hyperactivity/Inattention problems, Peer Relationship Problems, and Prosocial Behavior. It has parent- and teacher-rated versions for ages 4–16. The child-rated version is available for children aged 11–16 but its validity has been shown for younger children aged 4–11 (Norwood, 2007). Higher scores that are further from the standardized mean for emotional, behavior, hyperactivity/inattention, peer, and overall scales are indicative of greater reported difficulties. Lower scores on the prosocial scale are indicative of less prosocial behavior.

**Socioeconomic Status (SES).** SES was derived from occupation and employment status information, according to the National Statistics Socio-economic Classification (NS-SEC: Office for National Statistics, 2005). The self-coded version was used. The five levels of classification were (a) managerial and professional occupations, (b) intermediate occupations, (c) small employers and own account workers, (d) lower supervisory and technical occupations, and (e) routine occupations and unemployed.

### Statistical Analysis

Quantitative analysis was conducted using SPSS (version 17.0). First-step analysis assessed whether group means for cognitive and behavioral measures were lower for children following stroke. One-sample *t*-tests were conducted with the means from the participants of the childhood stroke group and compared to available standardized test norms. Secondly, standard scores from neuropsychological tests were transformed into *z*-scores using their normative mean and standard deviation. The FSIQ *z*-score was used to measure overall general intellectual functioning (“Intelligence” domain). A “Reading” domain score was computed by averaging *z*-scores from the two WIAT-II subtests. An “Attention” domain score was computed using the composite of TEA-Ch *z*-scores. An “Executive Function” domain score was computed by averaging the three sequencing subtests from the D-KEFS. BRIEF questionnaire scores were also transformed into *z*-scores. Third-step analysis involved examination of between-domain differences. For those focal areas identified as particularly vulnerable in the third step (i.e., those where significantly lower scores were obtained), within-domain differences were investigated at a fourth-stage analysis. Within-group differences between cognitive domains were assessed using repeated-measure analyses of variance (ANOVAs). Post hoc paired sample *t*-tests were used to identify specific differences. Finally, effects of age at injury were examined using partial correlations. Effect of hemispheric side of injury was examined using independent sample *t*-tests comparing those children with left- (*n* = 23) versus right-sided stroke (*n* = 21). Those with bilateral damage were excluded from the analysis due to small numbers (*n* = 5). Follow-up data were examined via T1-T2 comparisons using Wilcoxon Signed-rank.

## RESULTS

### Neuropsychological Profile Following Childhood AIS

As can be seen (Table 3), the mean scores for the group of children with stroke were significantly lower than standardized norms across all domains. Small-to-medium effect sizes were observed between mean scores for the childhood stroke group and standardized norms on domains of intelligence and academic achievement. Medium-to-large effect sizes were observed between the childhood stroke group and standardized norms on attention and executive function domains.

**Table 3:** Neuropsychological Measures for Clinical Sample Compared to Normative Means.

Domain	Measure	Variable	*n*	Test Population Mean (*SD*)	Sample Mean (*SD*)	Sample Range	*t*	*p*	Effect Size (Cohen's *d*)
General Intellect	WASI	FSIQ	49	100 (15)	92.06 (13.91)	60–118	−3.99	<.0001*	.5 (medium)
		VIQ	49	100 (15)	93.08 (13.86)	63–126	−3.5	.001*	.5 (medium)
		PIQ	49	100 (15)	93.61 (14.69)	64–132	−3.05	0.004	.4 (small-medium)
	WASI	Vocabulary	49	10 (3)	8.16 (2.97)	1–14	−4.33	<.0001*	.6 (medium)
	Subtests	Similarities	49	10 (3)	9.29 (2.90)	2–15	−1.72	0.091	.2 (small)
		Block Design	48	10 (3)	8.25 (2.91)	4–17	−4.16	<.0001*	.6 (medium)
		Matrix Reasoning	49	10 (3)	9.35 (3.11)	4–16	−1.47	0.148	.2 (small)
	WISC/WAIS Subtests	Digit Span	49	10 (3)	8.76 (3.53)	1–17	−2.47	.017*	.4 (small-medium)
		Coding	48	10 (3)	7.81 (2.74)	3–14	−5.53	<.0001*	.7 (medium-large)
Academic Attainments	WIAT	Reading Comprehension	47	100 (15)	93.02 (16.88)	61–126	−2.84	.007*	.5 (medium)
		Reading Speed	34	100 (15)	99.24 (16.11)	70–130	−0.28	0.784	.1 (small)
Attention	TEA-Ch	Sky Search Targets	46	10 (3)	8.74 (3.12)	4–15	−2.74	0.009	.4 (small-medium)
		Time Per Target	46	10 (3)	6.96 (3.01)	1–13	−6.86	<.0001*	1.0 (large)
		Attention Score	46	10 (3)	7.09 (3.19)	1–13	−6.2	<.0001*	.9 (large)
		Score	44	10 (3)	6.89 (3.73)	1–15	−5.54	<.0001*	1.0 (large)
		Sky Search Dual Task	42	10 (3)	4.07 (3.98)	1–14	−9.64	<.0001*	1.9 (large)
		Score Dual Task	41	10 (3)	7.20 (3.76)	1–16	−4.78	<.0001*	.9 (large)
		Walk/Don't Walk	41	10 (3)	5.37 (3.39)	1–14	−8.76	<.0001*	1.5 (large)
		Attention Composite Score	47	10 (3)	6.65 (2.37)	2.5–11	−9.68	<.0001*	1.1 (large)
Attention/ Executive	D-KEFS	Visual Scanning	34	10 (3)	8.76 (2.63)	1–13	−2.74	0.01	.4 (small-medium)
Function	Trail Making	Number Sequencing	34	10 (3)	8.00 (3.79)	1–14	−3.08	0.004	.7 (medium-large)
	Task	Letter Sequencing	33	10 (3)	7.30 (4.37)	1–14	−3.55	.001*	.9 (large)
		Letter Number Sequencing	33	10 (3)	7.48 (3.83)	1–14	−3.77	.001*	.8 (large)
		Motor Speed	33	10 (3)	10.42 (2.46)	1–14	0.99	0.33	.1 (small)

*Note*. *After correction for multiple comparisons, significant at *p* < .002.

To exclude the possibility that these results were disproportionately caused by the inclusion of children with moyamoya vasculopathy, all 10 children with moyamoya were excluded from a further exploratory analysis. Even with the moyamoya group excluded, the differences across all cognitive domains remained as outlined in Table 2, with the single exception of performance IQ on the WASI (*p* = .066).

Eighteen out of 44 participants changed handedness (40.1%) following their stroke. Exploratory analyses revealed that the group of children who had changed handedness since their stroke did not perform significantly differently from those who did not on any cognitive domain.

### Between-Domain Comparisons: Intelligence, Reading, Attention, and Executive Function

**Figure 1:**
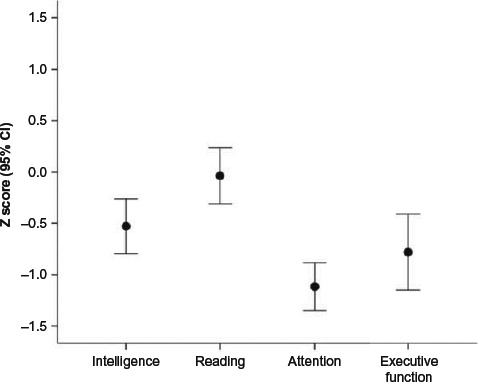
Outcome differences between domains of neuropsychological functioning.

All four composite z-scores fell within the lower end of the average range, with group means within one standard deviation of the normative sample (Figure 1). ANOVA revealed a significant effect of Domain, *F* = 10.73, *p* < .001, *η*_p_^2^ = .309, with post hoc comparisons indicating that both the Attention and Executive Function domains were significantly affected, relative to both Intelligence, *t* = 5.27, *p* < .0001 and *t* = 2.50, *p* = .018, respectively, and Reading, *t* = 5.41; *p* < .0001; *t* = 3.02, *p* = .006. Attention and executive function domains did not differ from each other (*p* > .20).

Linear regression analyses that included recurrent stroke/TIAs and a history of seizures as predictor variables and each of the four domains as outcome measures were conducted. Recurrent stroke/TIAs were not independently significant predictors for outcome on any of the four domains. History of seizures was an independent significant predictor of outcome on three of the four domains: General Intelligence, adj *R*^2^ = .113, *F* = 4.044, *p* < .05, *β* = .349, *p* < .05; Attention, adj *R*^2^ = .52, *F* = 2.250, *p* < .05, *β* = .320, *p* < .05; and Executive Function, adj *R*^2^ = .390, *F* = 11.221, *p* < .001, *β* = .652, *p* < .001.

### Within-Domain Vulnerabilities

**Figure 2:**
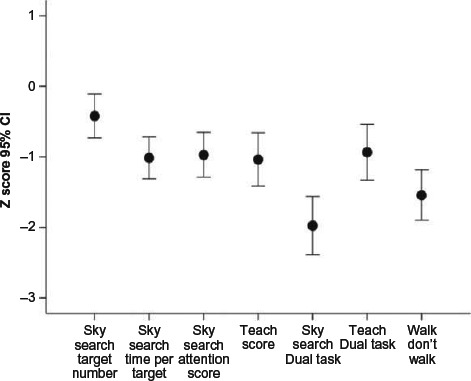
TEA-Ch subtest differences within the Attention domain.

Within the Attention domain, there was a significant main effect of subtest, *F* = 11.48, *p* < .0001. Only two subtest means from the TEA-Ch were very impaired, namely divided attention-dual modalities (TEA-Ch Sky Search Dual Task) and response inhibition (TEA-Ch Walk/Don't Walk subtest) (See Figure 2). Scores on these two subtests were significantly lower than those on the five other subtests (*p* < .0001 in all cases).

**Figure 3:**
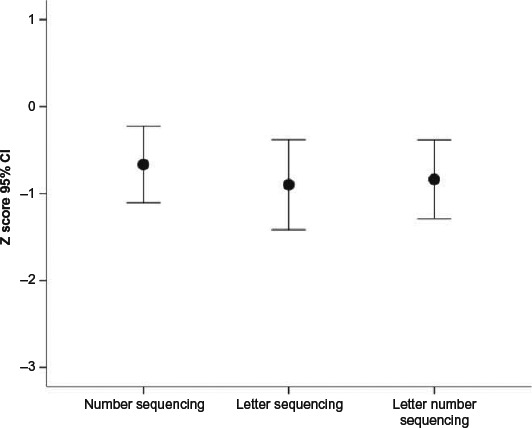
DKEFS subtest differences within the Executive Function domain.

Within the Executive Function domain (Figure 3), there were no significant differences between subtest scores, *F* = 0.87, *p* > .40.

### Frequency of Impairments in the Sample

To obtain an estimate of the frequency of impairments in our sample, participants with a *z*-score less than or equal to −1.5 standard deviations were classified as “Impaired,” as this is a commonly used cutoff in clinical settings. Using this categorization, 21/41 (51%) of participants were impaired on the response inhibition task (Walk/Don't Walk subtest of the TEA-Ch), while 28/42 (67%) were impaired on the dual-task subtest (Sky Search). On the executive function sequencing tasks, 10/33 were impaired (30%). The percentages were comparable (47%, 59%, and 22%, respectively) when participants with moyamoya disease were excluded from the sample.

### Behavioral Profile Following Childhood Stroke

Behavior and psychological questionnaire measures and the comparison with normative data are presented in Table 4.

**Table 4:** Psychological and Behavioral Measures for Clinical Sample Compared to Normative Means.

Domain	Measure	Variable	*n*	Test Population Mean (*SD*)	Sample Mean	Sample *SD*	*t*	*p*	Effect Size (Cohen's *d*)
Behavior	SDQ	Emotional	43	2.8 (2.1)	3.93	2.56	2.90**	.006	.5 (medium)
	Self ratings	Behavior	43	2.2 (2.2)	2.74	1.73	2.06	.046	.3 (small)
		Hyperactivity/Attention	43	3.8 (2.2)	3.84	1.94	0.13	.900	< .0 (−)
		Peer Problems	43	1.5 (1.4)	2.88	2.59	3.50**	.001	1.0 (large)
		Prosocial	43	8.0 (1.7)	7.33	2.12	−2.08	.043	.4 (small-medium)
		Impact	43	0.2 (0.8)	1.02	1.70	3.18	.003	1.0 (large)
		Total Overall Stress	43	10.3 (5.2)	13.40	6.22	3.26	.002	.6 (medium)
	SDQ	Emotional	49	1.9 (2.0)	3.37	2.81	3.64**	.001	.7 (medium-large)
	Parent ratings	Behavior	49	1.6 (1.7)	1.88	1.79	1.09	.282	.2 (small)
		Hyperactivity/Attention	49	3.5 (2.6)	4.86	2.92	3.26	.002	.5 (medium)
		Peer Problems	49	1.5 (1.7)	2.33	2.33	2.22	.012	.5 (medium)
		Prosocial	49	8.6 (1.6)	8.33	1.75	−1.10	.279	.2 (small)
		Impact	47	0.4 (1.1)	1.85	2.74	3.63**	.001	1.3 (large)
		Total Overall Stress	49	8.4 (5.8)	12.43	7.49	3.77**	<.0001	.7 (medium-large)
	SDQ	Emotional	41	1.40 (1.9)	2.05	2.21	1.88	.068	.3 (small)
	Teacher ratings	Behavior	41	0.9 (1.6)	0.83	1.40	−0.33	.747	< .0 (−)
		Hyperactivity/Attention	41	2.9 (2.8)	3.46	2.51	1.44	.159	.2 (small)
		Peer Problems	41	1.4 (1.8)	1.27	2.03	−4.16	.679	.1 (small)
		Prosocial	40	7.2 (2.4)	7.80	2.31	1.64	.109	.3 (small)
		Impact	39	0.4 (1.0)	0.54	0.88	0.98	.334	.1 (small)
		Total Overall Stress	41	6.6 (6.0)	7.61	5.50	1.18	.247	.2 (small)
Executive Function	BRIEF	BRI	19	50 (10)	52.53	9.85	1.12	.278	.3 (small)
	Self	MI	19	50 (10)	51.16	9.54	0.53	.603	.1 (small)
	Ratings (11+)	GEC	19	50 (10)	52.00	9.89	0.88	.390	.2 (small)
	BRIEF	BRI	49	50 (10)	57.63	15.89	3.36	.002	.8 (large)
	Parent	MI	48	50 (10)	56.29	13.32	3.27	.002	.6 (medium)
	ratings	GEC	48	50 (10)	57.27	14.60	3.45**	.001	.7 (medium-large)
	BRIEF	BRI	40	50 (10)	56.18	13.03	2.94	.006	.6 (medium)
	Teacher	MI	40	50 (10)	60.63	14.11	4.76**	< .0001	1.1 (large)
	ratings	GEC	40	50 (10)	59.8	13.75	4.51**	< .0001	1.0 (large)

*Notes.* BRI = Behavioral Regulation Index; MI = Metacognitive Index; GEC = Global Executive Composite. Higher scores, further from standardized norms, on SDQ and BRIEF are indicative of greater difficulties, with the exception SDQ prosocial behavior, where lower scores are indicative of less prosocial behavior.

** Significant at *p* ≤ .001.

As measured by the SDQ, emotional functioning and overall impact on life were rated by children and their parents as areas of difficulty. In addition, children also rated increased difficulties with peers. Parents perceived hyperactivity as problematic. Teachers’ ratings did not differ significantly from normative data. Young person's ratings (for those aged 11 and older; *n* = 19) on a questionnaire of everyday executive function behavior (BRIEF) did not differ significantly from population norms across any executive function domain. In contrast, all parent-rated and all teacher-rated BRIEF overall index scores (Behavioral Regulation, Metacognitive Index, and Global Executive Composite) were significantly higher than age-scaled norms, indicating that parents and teachers both identified significant behavioral difficulties in global behavioral executive function abilities.

**Figure 4:**
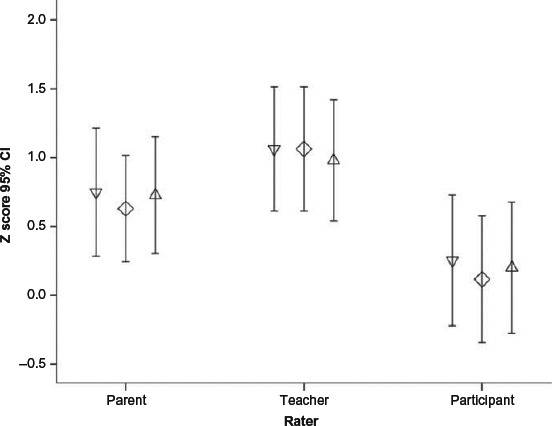
BRIEF scores across domains and raters, where higher scores denote more difficulties. Inverted triangles = Behavioral Regulation Index (BRI); Diamonds = Metacognition Index (MI); Triangles = Global Executive Composite (GEC).

A mixed-model ANOVA comparing the scores from the three groups of raters (children, parents, teachers) across the three BRIEF subdomains (BRI, MI, and GEC) revealed a trend for a significant effect of Rater, *F* = 2.62, *p* = .078 (see Figure 4). Young people's ratings were significantly below those of the teachers for BRI, *t* = −2.25, *p* = .028, MI, *t* = −2.65, *p* = .01, and GEC, *t* = −2.2, *p* = .03.

Correlational analysis was conducted to compare child-, parent-, and teacher-rated BRIEF to performance on attention and executive function domains. As Table 5 indicates, several parent and teacher ratings on the BRIEF were significantly correlated with attention and executive function domains. Performance on executive function domains, but not attention, were significantly associated with child-rated BRIEF.

**Table 5:** Correlation Between Neuropsychological and Questionnaire Data.

BRIEF	Attention Domain	Executive Function Domain
Child-rated		
BRIEF	−.33	−.473*
BRI	−.267	−.521*
MI	−.298	−.540*
GEC		
Parent-rated		
BRI	−.439**	−.371*
MI	−.405**	−.281
GEC	−.438**	−.34
Teacher-rated		
BRI	−.129	−.254
MI	−.356*	−.521**
GEC	−.302	−.442*

### Effects of Lateralization and Age of Stroke on Outcome

**Effect of Hemispheric Side of Stroke.** There was no significant difference between the left and right hemisphere groups on variables including age at stroke, age at assessment, time since stroke, neurological severity, history of seizures, SES (independent sample *t*-tests, *p* > .15 in all cases).

There were no significant differences between the left and right hemisphere stroke groups on any of the broad cognitive domains or BRIEF scores. The only significant difference across all the specific subtests was found on the Matrix Reasoning subtest of the WASI, with the group who experienced right-sided stroke performing significantly better (*M* = 10.62, *SD* = 2.5) than the left-sided group (*M* = 8.65, *SD* = 3.35), *t*(42) = −2.189, *p* < .034.

**Effect of Age at Stroke.** There were no significant associations between age at stroke and intelligence (*r* = −.247, *p* = .088), reading (*r* = .254, *p* = .147), or attention domain scores (*r* = .092, *p* = .538). For the executive function domain, earlier age of stroke was significantly associated with better performance (*r* = −.36, *p* = .041). There were no significant associations between age at assessment and any of the cognitive domains. On the self-rated BRIEF, older age of stroke was associated with more self-rated difficulties (Correlations with age at stroke: BRI: *r* = .59, *p* = .007; MI: *r* = .58, *p* = .009; GEC: *r* = .62, *p* = .005). Age at stroke was significantly associated with both age at assessment (*r* = .566, *p* < .001) and time since stroke (*r* = −.469, *p* = .001). Age at assessment was also significantly correlated with time since stroke (*r* = .462, *p* = .001). Regression analyses were conducted to explore whether age at stroke and age at assessment were significant predictor variables for those executive function measures that were found to be significantly correlated. While age at stroke remained a significant predictor on the self-rated BRIEF, age at assessment was not a significant predictor of BRI, MI, or GEC or for the executive function domain. For the executive function domain score, age at stroke was a significant independent predictor, adjusted *R*^2^ = .10, *F* = 4.56, *p* = .041, standardized *β* = −0.36. When both age at stroke and age at assessment were included as predictor variables in the model, then age at stroke was no longer a significant predictor of outcome, adjusted *R*^2^ = .07, *F* = 2.2, *p* = 0.12.

**Longitudinal Follow-Up Study.** There were no significant differences between group means at initial assessment and follow-up for intelligence, attention, and executive function, indicating that children's performance on neuropsychological assessment did not change over time. Parent and child reports at T1 and T2 demonstrated that children's levels of emotional functioning and overall impact on life also did not change significantly over time (see Table 6).

**Table 6:** Neuropsychological, Psychological, and Behavioral Measures for Clinical Sample at T1-T2.

Domain	Measure	Variable	T1 *n*	Test Population Mean (*SD*)	T1 Sample Mean (*SD*)	Sample Range	T2 *n*	T2 Sample Mean (*SD*)	Sample Range	*z*	*p*
General Intellect	WASI	FSIQ	9	100 (15)	87.33 (11.81)	76–113	9	91.11 (17.35)	73–127	−1.30	.19
		VIQ	9	100 (15)	91.88 (13.34)	75–119	9	94.44 (14.97)	76–123	−0.54	.59
	WASI Subtests	PIQ	9	100 (15)	88.44 (9.83)	74–103	9	89.44 (17.95)	67–124	0.00	1.00
		Vocabulary	9	10 (3)	7.88 (3.25)	5–14	9	8.78 (3.93)	4–16	−0.20	.32
	WISC/ WAIS	Similarities	9	10 (3)	9.11 (2.71)	5–14	9	9.33 (2.65)	6–14	−0.63	.53
	Subtests	Block Design	9	10 (3)	6.88 (2.08)	4–11	9	7.33 (3.60)	3–14	−0.86	.39
		Matrix Reasoning	9	10 (3)	8.66 (2.23)	5–12	9	8.44 (3.84)	4–14	−0.42	.67
		Digit Span	9	10 (3)	6.55 (1.81)	3–9	9	4.89 (3.79)	0–11	−1.27	.20
		Coding	9	10 (3)	6.88 (2.20)	3–10	9	5.11 (4.01)	0–10	−1.20	.23
Attention	TEA-Ch	Sky Search Attention Score	8	10 (3)	5.75 (3.28)	1–12	7	5.43 (4.12)	1–12	−0.27	.78
		Score	9	10 (3)	5.00 (2.64)	1–10	6	5.67 (4.55)	1–13	−0.41	.68
		Sky Search Dual Task	8	10 (3)	2.75 (1.90)	1–6	6	4.83 (4.49)	1–11	−1.29	.20
		Score Dual Task	8	10 (3)	5.50 (3.16)	1–9	6	5.83 (1.60)	4–8	−1.13	.26
		Walk/ Don't Walk	9	10 (3)	2.77 (2.10)	1–6	6	3.50 (3.73)	1–10	−0.37	.72
Executive	BRIEF	BRI	8	50 (10)	68.2 (9.17)	52–82	8	61.50 (8.75)	45–74	−0.42	.67
Function	Parent ratings	MI	8	50 (10)	64.75 (12.06)	45–80	8	62.75 (11.49)	43–78	−0.31	.72
		GEC	8	50 (10)	67.00 (10.23)	48–80	8	62.88 (9.69)	50–78	−0.11	.92
Behavior	SDQ	Emotional	7	2.8 (2.1)	3.42 (2.30)	1–8	6	3.67 (3.61)	0–8	−0.37	.71
	Self ratings	Behavior	7	2.2 (2.2)	3.14 (1.77)	1–6	6	1.83 (2.04)	0–4	−2.06	.04
		Hyperactivity/Attention	7	3.8 (2.2)	3.29 (2.09)	1–7	6	3.17 (1.83)	0–4	−0.11	.92
		Peer Problems	7	1.5 (1.4)	1.57 (1.72)	0–4	6	1.67 (1.03)	0–3	−0.32	.75
		Prosocial	7	8.0 (1.7)	7.14 (2.19)	5–10	6	7.00 (3.58)	0–10	−0.37	.72
		Impact	7	0.2 (0.8)	2.00 (2.38)	0–6	6	0.83 (1.32)	0–3	−1.20	.27
		Total Overall Stress	7	10.3 (5.2)	11.43 (5.13)	7–21	6	10.33 (6.97)	0–18	−0.52	.60
	SDQ Parent ratings	Emotional	8	1.9 (2.0)	4.88 (2.23)	1–7	7	2.86 (2.12)	0–5	−0.95	.34
		Behavior	8	1.6 (1.7)	2.25 (1.28)	0–4	7	1.86 (1.35)	0–4	−1.13	.26
		Hyperactivity/Attention	8	3.5 (2.6)	5.75 (1.83)	3–8	7	4.43 (1.99)	2–8	−1.86	.06
		Peer Problems	8	1.5 (1.7)	2.88 (2.23)	0–7	7	3.29 (2.43)	0–7	−1.29	.20
		Prosocial	8	8.6 (1.6)	8.63 (1.85)	5–10	7	8.14 (1.35)	6–10	−0.38	.70
		Impact	8	0.4 (1.1)	3.25 (1.58)	1–5	7	8.14 (1.35)	6–10	−1.36	.18
		Total Overall Stress	8	8.4 (5.8)	15.75 (3.37)	10–20	7	10.71 (7.34)	0–20	−0.94	.35

## DISCUSSION

### Vulnerabilities in Attention and Executive Functions

Following AIS, performance as a group was significantly lower than standardized norms across all cognitive domains assessed. However, particular vulnerabilities were found in attention and executive function domains, beyond the mild reductions in general intellectual abilities and academic attainments. Particular weaknesses were found in divided attention across dual modalities (auditory and visual) and for response inhibition. Half the sample was classified as “Impaired” (> 1.5 standard deviation below mean) on a task of response inhibition and two thirds were classified as “Impaired” on a dual modality divided attention task. Difficulties were also highlighted with sequencing, switching, working memory, and cognitive flexibility. Just over a quarter of the sample were classified as “Impaired” on these tasks. These findings support the view that widespread neural involvement is crucial for executive functions in the developing brain, as the current cohort had predominantly basal ganglia and MCA infarcts, with predominantly spared cortical frontal lobes. History of seizures poststroke was a significant predictor of poorer performance on general intelligence, attention, and executive function measures overall. Recurrent strokes or TIAs were not significant predictors of these domains. There was no evidence that motor performance or changed handedness was related to cognitive performance on the assessments.

### Behavior and Emotional Regulation Ratings

A large proportion of parents (up to 69%) have reported concerns about their child's behavior in previous research (Ganesan et al., 2000; Pavlovic et al., 2006; Steinlin et al., 2004). Using a triangulation approach by comparing ratings reported by children, parents, and teachers with standardized neuropsychological assessments was informative. Consistent with the neuropsychological assessment scores, parents and teachers, but not children themselves, identified significant difficulties in the areas of behavioral regulation, metacognitive skills, and global everyday executive function abilities. The relationships observed were strongest between performance on tests and parent reports of difficulties with metacognition, whereas, for teachers, associations were identified between deficits with executive function and behavioral regulation. The reasons for these associations are not clear but may relate to the various demands of different environments on the child (e.g., school vs. home and a parent who was aware of the child's abilities preinjury vs. a teacher who has known the child for a shorter period of time). This triangulation approach also allowed us to identify areas of weaknesses the children themselves are not aware. As executive function skills and self-awareness continue to develop throughout adolescence (Blakemore & Choudhury, 2006), impairments may be particularly difficult for children to recognize following ischemic stroke. However, as the self-rated BRIEF is validated for children aged 11 and older, only a subsample (*n* = 19) of the larger group completed this questionnaire. Therefore, these findings relating to children's insight into their deficits must be interpreted with caution due to the relatively low sample size.

### General Intellectual Functioning and Academic Attainment

Consistent with previous research (Anderson et al., 2009; Long, Anderson, et al., 2011; Westmacott et al., 2009), general intellectual functioning fell within the average range overall but was significantly lower than standardized norms, with a large range of scores. Similar findings were observed with reading comprehension abilities and highlight the importance of careful monitoring over time of academic abilities within the school context following childhood stroke.

### Longitudinal Follow-Up Study

Longitudinal follow-up findings of a smaller subgroup demonstrated that the cognitive abilities of children who experienced ischemic stroke at least 2 years previously remains stable over time. This suggests that those vulnerable neuropsychological areas in general intellect, academic attainment, attention, and executive function are relatively consistent in the longer term.

### Effect of Age of Stroke on Executive Function

Earlier age of stroke was associated with better performance on executive function tasks of sequencing and switching and with fewer self-rated everyday executive function behavioral difficulties. This is an unexpected and interesting finding, as attentional control skills have been reported as the first executive skills to emerge and perhaps most vulnerable to early insult (Anderson, Catroppa, Morse, Haritou, & Rosenfeld, 2005; Long, Spencer- Smith, et al., 2011). One possibility, supporting the early plasticity hypothesis, is that older age-of-stroke onset results in more disruption to circuitry developing at a later stage and is associated with poor development of cognitive flexibility (D-KEFS sequencing and switching) and more self-rated everyday executive function skills. A staged process of development of higher order association cortices has been shown. It is possible that different aspects of cognition may be vulnerable at different developmental stages (Gogtay et al., 2004; Shaw et al., 2008). Long, Spencer-Smith, et al. (2011) found that earlier age of stroke onset was associated with poorer performance on some executive function tasks but with better performance on another executive function task of goal setting. The effect of age of onset may not be a linear relationship. There may be multiple factors involved, including specific domains of cognition being vulnerable at different developmental periods (Allman & Scott, 2013; Anderson et al., 2009; Westmacott et al., 2009), lesion size (Long, Anderson, et al., 2011), cortical and subcortical involvement (Long, Spencer-Smith et al., 2011), stroke recurrence, seizure activity (Royal College of Physicians of London, 2004), changes in metabolism postbrain injury (Prins, Alexander, Giza, & Hovda, 2013). Anderson, Spencer-Smith, and Wood (2011) also describe a “recovery continuum” with various individual, injury, environmental, and intervention factors that can influence outcome. Given that three age-related factors—age at stroke, age at assessment, and time since stroke—are correlated with each other in the present study, it is clear that these factors are difficult to disentangle from each other in cross-sectional research (Taylor & Alden, 1997). However, age at stroke remained an independent predictor of self-rated executive function behavior, even after including age at assessment in the model. While further research is necessary to clarify this issue, the results from the current study nonetheless offer some support for the early plasticity hypothesis, at least for some executive function tasks. A further factor in the present study is that this group of children did not include anyone who had experienced a stroke in the neonatal or perinatal period. Many previous studies include a more heterogeneous group including neonatal, perinatal, and childhood stroke (e.g., Max et al., 2004; Pavlovic et al., 2006; Westmacott et al., 2009). Westmacott et al. found that their perinatal group performed more poorly than the older groups on most cognitive measures, regardless of lesion location. It is, therefore, a possibility that previous studies that have included prenatal, neonatal, and perinatal strokes with strokes in later childhood have impacted their outcome findings.

### Effect of Hemispheric Side of Stroke

The lack of lateralization effects found here is consistent with previous studies with children that failed to find the well-documented lateralized linguistic, cognitive, and emotional differences reported in adulthood following left and right hemisphere stroke (Long, Spencer-Smith, 2011; Max, 2004;). However, participants with left hemisphere stroke performed more poorly on a task of nonverbal functioning. Allman and Scott (2013) reported that children with left-sided cortical stroke performed worse on several neuropsychological measures, including working memory, delayed verbal memory, and receptive language. In the current study, at least half the sample had subcortical involvement, which may be indicative of more diffuse regions affected and may have potentially limited identification of any lateralization effects. Executive and attention functions assessed here (response inhibition, dual attention, sequencing) are likely to be represented in widespread networks, yet vulnerable to unilateral injury. Therefore, integrity of both left- and right-sided networks may be necessary for the normal development of these functions. Further exploration of any lateralization effects with a larger data set is warranted.

### Clinical Implications

The specific vulnerability of attention, executive functions, and emotional regulation in our sample indicates that screening assessments are essential for *all* children following childhood ischemic stroke to ensure that specific difficulties with higher level cognitive abilities that could impact learning and academic achievement are not missed over time. As in Anderson et al. (2011), the full extent of cognitive and behavioral consequences for children following AIS may not be apparent until many years after the initial insult. Neuropsychological assessments would benefit from including standardized measures of attention, executive function, behavioral, and psychological measures. The triangulation of various sources (parent, teacher, individual) and questionnaires (individually administered, self-report ecologically valid questionnaire report) of information is very important in understanding how the effects of stroke may impact the child's life and which contexts may be best suited to intervention. Furthermore, the longer term follow-up data in this study indicated that the deficits persist over time. Targeted cognitive and psychosocial interventions should be evaluated to assess their impact on improving the lives of children who have experienced stroke. Cognitive rehabilitation that has proven successful with children following acquired brain injury should be investigated for their efficacy with childhood stroke (Butler & Copeland, 2002; Galbiati et al., 2009; Laatsch et al., 2007; Marcantuono & Prigatano, 2008; Van't Hooft et al., 2005; see Ross, Dorris, & McMillan, 2011 for review).

### Limitations

In interpreting the findings from the current study, several limitations need to be considered. Firstly, this study did not have a control group from which to compare the findings. This meant that analysis was reliant on comparing to standardized norms. Although relatively common in the childhood stroke literature (e.g., Almann & Scott, 2013; Westmacott et al., 2009; Pavlovic et al., 2006; Long, Anderson, et al., 2011; Long, Spencer-Smith, et al., 2011), a concurrently recruited appropriate comparison group would have strengthened the conclusions. However, there are also several issues inherent in the selection of an appropriate control group for childhood neurological research. Age- and sex-matched controls may not be representative in terms of SES, intellectual ability, or ethnicity. Healthy siblings may not be age and sex matched. Medical controls, such as those with sickle cell disease (SCD) or moyamoya disease but without history of stroke, allow for matching of factors related to chronic health conditions. However, SCD itself may be associated with subtle cognitive effects, even for children without stroke (Max et al., 2003; Schatz, Craft, Koby, & DeBaun, 2004). Williams et al. (2012) found that children with moyamoya vasculopathy are also at risk for intellectual and executive function difficulties, regardless of history of stroke or silent stroke. Ideally, perhaps neurologically healthy age-, sex-, and SES- matched controls *and* a group of children with physical disabilities may be best to recruit as control groups for future research in order to isolate the neurological and cognitive components with the childhood stroke group (as in Max et al., 2004).

Secondly, as is the case with many neuropsychological studies, the different normative groups used in the standardization of assessments may be relevant when interpreting the relative magnitude of the observed deficits across domains. For example, different standardized normative data are used in the TEA-Ch, WASI, and D-KEFS. However, this study compared both between as well as within domains.

Thirdly, limitations inherent in cross-sectional research in developmental neuropsychology may also be relevant, as three age-related factors (age at stroke and assessment and time since stroke) were interrelated in the current study. Longitudinal, prospective follow-up studies from acute period to long-term follow-up would help to clarify some of the issues regarding age-related factors (Taylor & Alden, 1997). Future research that includes functional neuroimaging may also help to clarify and overcome many of these issues relating to vulnerability, plasticity, and reorganization of the developing brain following stroke.

Fourthly, although we carefully selected our large sample to maintain homogeneity by recruiting only those who experienced AIS in childhood and limiting the age range to 6 years and older, there was nonetheless heterogeneity and variability in risk factors (e.g., history of TIAs or stroke recurrence, history of seizures), physical disability, and outcome. In the current study, history of seizures was a significant predictor of outcome on neuropsychological measures but stroke recurrence and changed handedness were not. This variability in outcome may be indicative of a true representation of young people post-AIS who present at specialist clinics. Ideally, large, multisite collaboration and recruitment would be helpful to confirm our findings in a wider population sample. This would allow for careful selection of subgroups within the larger samples that could further explore the impact of risk factors including stroke recurrence and seizure activity on cognition and behavioral outcome. Furthermore, larger samples from any such multisite collaboration would allow for further cognitive and behavioral profiling that may be associated with different etiologies of childhood AIS, such as sickle cell disease and moyamoya syndrome. This may also allow for more specific lesion or brain region comparisons, such as cortical versus subcortical, and to explore any lateralization effects further or frontal versus non-frontal involvement.

### Conclusions

This study is the first to include a triangulated data from children, parents, and teachers for ratings of behavior and executive function following AIS. This large cohort indicates that attention, executive function, and emotional regulation are significantly impacted following childhood AIS, beyond the mild reductions in general intellectual and academic abilities. These executive functioning difficulties are clearly evident to parents and teachers but may not be identified by children themselves. This study also supports the view that unilateral disruption of diffuse networks in the developing brain can lead to impaired executive function skills. Finally, the study demonstrates that, following AIS, the cognitive profile of children remains stable over time, highlighting the need for appropriate interventions to support this group.
